# Supplementary material for: l-Alanine Exporter AlaE Functions as One of the d-Alanine Exporters in Escherichia coli
Source: Int J Mol Sci. 2023 Jun 16;24(12):10242. doi: 10.3390/ijms241210242 (PMC10299036; doi:10.3390/ijms241210242)
Supplement: Supplementary file 1 [file ijms-24-10242-s001.zip › Figure_S1.pdf]

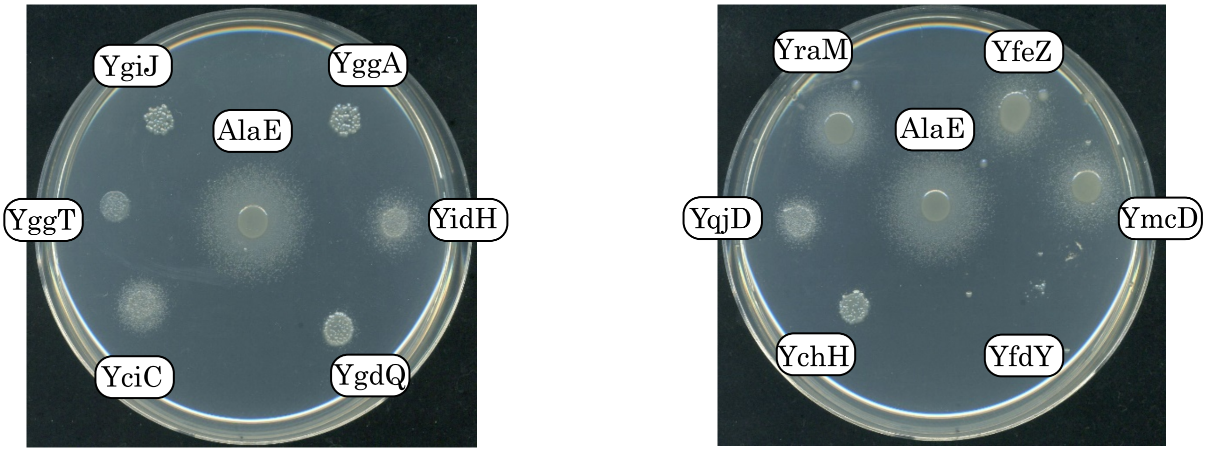

Figure S1. Representative result of the first screening. Overnight-grown cells of selected ASKA clones were washed twice with 0.85% NaCl and suspended in the original volume of the same solution. Subsequently, 5 μL of each clone was spotted on LB agar medium impregnated with the indicator strain *E. coli* MB2795 under the conditions of 0.01 mM IPTG and 6 mM Ala-Ala. The screening plates were incubated overnight at 37°C.
